# Developmental features of cotton fibre middle lamellae in relation to cell adhesion and cell detachment in cultivars with distinct fibre qualities

**DOI:** 10.1186/s12870-017-1017-3

**Published:** 2017-03-31

**Authors:** Mercedes C. Hernandez-Gomez, Jean-Luc Runavot, Frank Meulewaeter, J. Paul Knox

**Affiliations:** 1 Centre for Plant Sciences, Faculty of Biological Sciences, University of Leeds, Leeds, LS2 9JT UK; 2 Bayer CropScience NV - Innovation Center, Technologiepark, 38, 9052 Ghent, Belgium

**Keywords:** Cotton fibre, Middle lamella, CFML, Cell adhesion, Cell wall, *Gossypium* spp., Polysaccharides

## Abstract

**Background:**

Cotton fibre quality traits such as fibre length, strength, and degree of maturation are determined by genotype and environment during the sequential phases of cotton fibre development (cell elongation, transition to secondary cell wall construction and cellulose deposition). The cotton fibre middle lamella (CFML) is crucial for both cell adhesion and detachment processes occurring during fibre development. To explore the relationship between fibre quality and the pace at which cotton fibres develop, a structural and compositional analysis of the CFML was carried out in several cultivars with different fibre properties belonging to four commercial species: *Gossypium hirsutum*, *G. barbadense*, *G. herbaceum* and *G. arboreum*.

**Results:**

Cotton fibre cell adhesion, through the cotton fibre middle lamella (CFML), is a developmentally regulated process determined by genotype. The CFML is composed of de-esterified homogalacturonan, xyloglucan and arabinan in all four fibre-producing cotton species: *G. hirsutum, G. barbadense, G. herbaceum* and *G. arboreum*. Conspicuous paired cell wall bulges are a feature of the CFML of two *G. hirsutum* cultivars from the onset of fibre cell wall detachment to the start of secondary cell wall deposition. Xyloglucan is abundant in the cell wall bulges and in later stages pectic arabinan is absent from these regions.

**Conclusions:**

The CFML of cotton fibres is re-structured during the transition phase. Paired cell wall bulges, rich in xyloglucan, are significantly more evident in the *G. hirsutum* cultivars than in other cotton species.

## Background

Cotton fibres are single-cells and individual fibres start elongating from the seed surface as separate entities. The fibres then adhere together for the fibre elongation phase and detach again during later stages of fibre development. This makes cotton fibre cells an exceptional model to study cytokinesis-independent processes of plant cell adhesion and cell detachment as such processes are rarely present in the same developmental system. Cotton fibre cell development is a very finely regulated process which commences at the day of anthesis and commonly lasts between 50 and 60 days. Fibre development is usually divided into five sequential and overlapping stages: initiation, elongation, transition, secondary cell wall synthesis and desiccation (often misleadingly referred as maturation).

At the initiation stage (from 0 to 3–5 dpa) epidermal cells arise from certain cells at the seed surface with fibre initials and non-fibre cells in a 1:3.7 ratio [1]. One seed can generate approximately 14,500 lint (long) fibres [2], giving a fibre density of up to 1300 fibres/mm^2^ [3]. Considering that the flower ovary encloses 4 to 5 carpels (locules) which commonly contain 8 seeds (ovules) each it has been hypothesized that cotton fibres become adhered as a requirement in the highly packed environment inside the locule so that space can be optimised and high turgor pressure maintained during a coordinated fibre elongation phase. At this stage cotton fibres acquire a conical tip shape and elongate in adhered groups in a spiral-like manner [3, 4].

The matrix of polymers between two adhered plant cells is referred to as the middle lamella and the cotton fibre middle lamella (CFML) was first described by Singh et al. [4] in *Gossypium hirsutum*, one of the most commercialised species for fibre production. The CFML is a specialized outer layer of primary cell walls that mediates cell adhesion and links several fibres together into tissue-like bundles. The CFML therefore does not originate from the cell plate formed after cytokinesis but upon cell contact between cells at the beginning of the cell elongation phase. This is developmentally distinct from what is commonly considered as plant cell middle lamellae that mediate cell adhesion between daughter cells and that is established from cell plates during cytokinesis [5–9]. Similarly to the cell-plate-derived middle lamellae, the CFML was found to be rich in xyloglucan and de-esterified homogalacturonan polysaccharides using monoclonal antibodies [4]. As fibres elongate, they turn and fold over themselves in a grouped and ordered manner due to the adhesive properties of the CFML until the start of secondary cell wall deposition [4].

Cotton fibres do not stop elongating abruptly but undergo a transition phase characterized by an increase in the rate of cellulose synthesis [10] and the formation of the winding layer (similar to the S1 layer in xylem vessels) which is the first of the secondary cell wall layers to be deposited internal to the primary cell wall [11]. It is at this stage that fibres detach from each other at the onset of the major phase of secondary cell wall deposition. The timing of these developmental events depends on the genotype and is also influenced by temperature and environmental conditions during fibre growth [12–15]. Cotton fibre length is a major trait for the textile and derivative industries. Most of the fibre elongation occurs once cells are adhered and before they are detached from each other and secondary cell walls start being deposited. The amount of time that fibres are in the elongation phase partly determines final fibre length. Therefore understanding the mechanisms behind CFML mediation of cell adherence and detachment is important knowledge with the potential to be applied to the improvement of fibre quality.

Many aspects of the CFML remain to be uncovered, such as a comprehensive understanding of its structure and composition and a comparative analysis of the developmental timing for cell adhesion and detachment processes through the CFML in different cotton genotypes. These questions have been addressed here and the different timings of cell adhesion and cell detachment between genotypes with very different fibre qualities (fibre quality parameters described in [16]) have been determined. Moreover, two distinctive cell wall structural features of the CFML in *G. hirsutum* cultivars have been identified which may be determinants of the extent of fibre cell elongation in this species. Using immunochemistry techniques we have identified the polysaccharide arabinan to be part of the CFML in addition to pectic HG and xyloglucan. Taken together these results suggest that the timings of cell adhesion and cell detachment mediated by the CFML are different between genotypes, potentially affecting fibre quality traits.

## Methods

### Plant materials

The plants, and associated fibre properties, used in this study were the same as those described [16]. In short, seeds from six domesticated inbred cotton lines (FM966 and Coker312 - *G. hirsutum*, JFW15 - *G. arboreum*, Krasnyj - *G. herbaceum*, China10 and PimaS7 - *G. barbadense*) were sown in soil compost to which 30 g of Osmocote® 11 N + 11P + 18 K+ 2MgO + TE (working duration 5–6 months) was added to each pot and grown at Bayer Cropscience, Ghent. The settings for the day temperature were 24–26 °C and temperature increased with warm weather up to 30–35 °C with a 16 h photoperiod, except when outside light was >0.25 μmol m^−2^ s^−1^. All cotton flowers were tagged on the day of anthesis so that bolls could be harvested at the desired dpa. In one case ovule culture was used. Cotton ovaries were harvested at 1 or 2 dpa and sterilized in 70% ethanol for 5 min and washed with sterile water. All ovules of same ovary were transferred to a Petri dish under sterile conditions. Each Petri dish contained 12 ml of media (MS medium 4 (M0238), macronutrients (50 mM KNO3 as unique nitrogen source, 2 mM KH2PO4, 2 mM MGSO4), vitamins (4 μM nicotinic acid, 4 μM pyridoxine-HCl, 4 μM thiamine-HCl, 1 μM myo-inositol), pH 5, 100 mM glucose, 20 mM fructose, 10 μM IAA and 0.5 μM GA_3_). Ovules were cultured in the dark at 28 °C and media was changed every 3–5 days.

### Resin-embedding of plant materials

Immediately after collection, small regions of fibre tissues were carefully dissected from cotton bolls, causing minimal tissue disruption, and submerged in 4% (*v*/v) paraformaldehyde in PEM (0.1 M PIPES pH 6.95, 2 mM EGTA, 1 mM MgSO4) buffer. Fixed developing fibres were dehydrated, resin-embedded and sectioned as described previously [17].

### Monoclonal antibodies (mAbs)

Monoclonal antibodies used in this study: LM15 [18], LM25 [19], CCRC-M1 [20], LM6 [21–23], LM13 [24, 25] and LM19 [26].

### Microscopy and immunochemistry procedures

Resin sections were used for immunofluorescence detection of cell wall epitopes. PBS with 5% (*w*/*v*) milk protein was added for 30 min at room temperature to prevent non-specific binding. Primary antibodies were used at a 1:5 dilution (except for directly coupled LM19, see below, which was used at 1:1000) in 5% milk/PBS for 1.5 h. Goat anti-rat, anti-mouse or ant-rat IgG Alexa Fluor488 (Life Technologies) were used as secondary antibodies in a 1:100 dilution in 5% milk/PBS and samples were incubated for 1 h. Calcofluor White (Sigma–Aldrich) was used at 0.02 mg/ml in PBS for 5 min for visualization of cell walls. Anti-fade reagent Citifluor glycerol/PBS (Agar Scientific) was used to mount slides. Immunofluorescence imaging was performed using an Olympus BX61 microscope (http://www.olympus-global.com/) equipped with epifluorescence irradiation. Micrographs were obtained with a Hamamatsu ORCA285 camera (Hamamastu, http://www.hamamatsu.com) and PerkinElmer Volocity software. All related and comparative micrographs were captured using equivalent settings, and relevant micrographs were processed in equivalent ways for the generation of datasets. In all cases the area shown in each micrograph is representative of the fibre tissue seen in at least 3 sections analysed from one plant. In the case the FM966 line the micrographs are representative of the analysis of more than 3 plants.

## Results

### Two distinctive features of cell walls/CFML in cotton fibre tissues

**Fig. 1:**
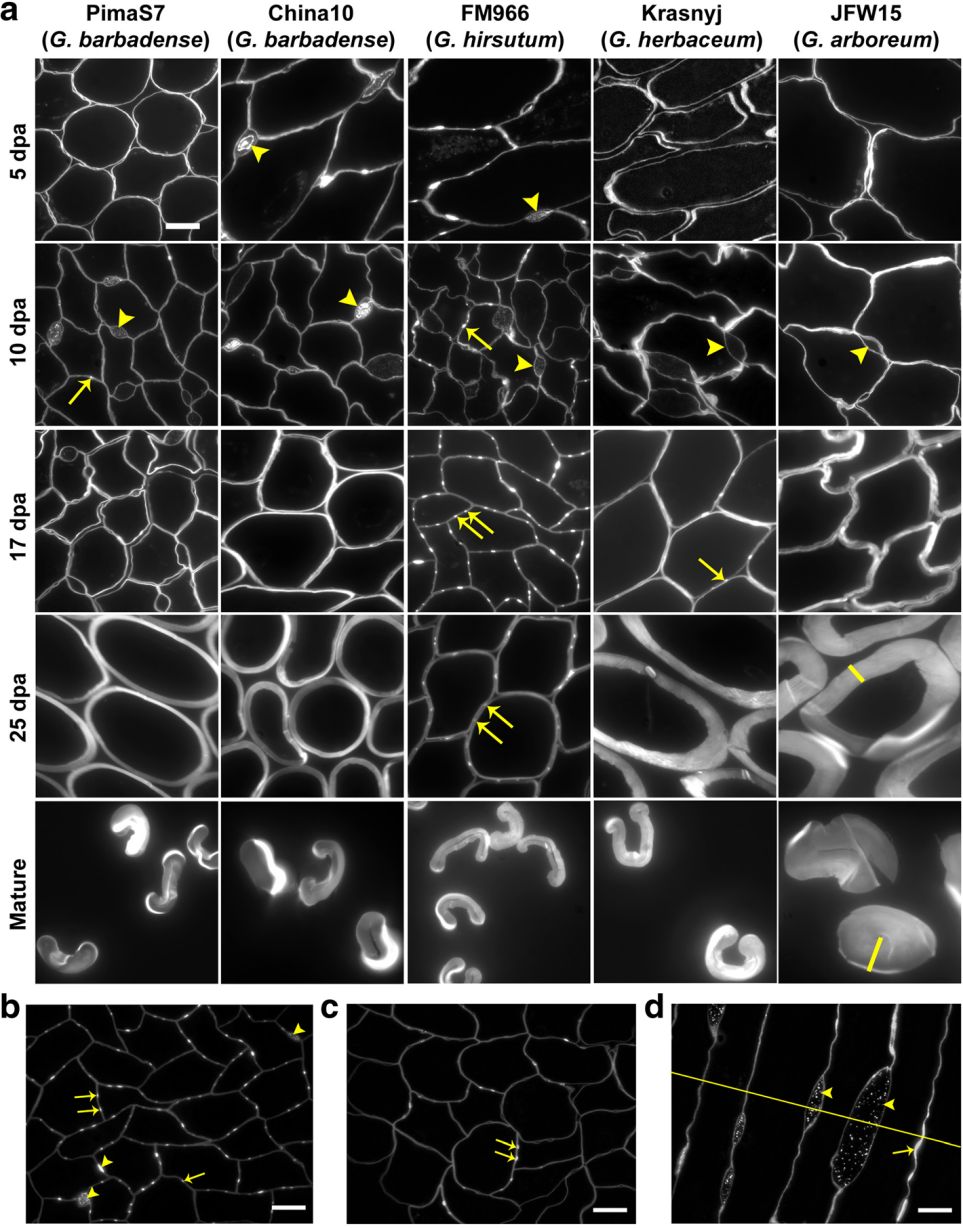
Variation of the developmental features of the CFML by genotype. **a** Calcofluor staining of cross sections of developing and mature cotton fibres of PimaS7 and China10 (*G. barbadense*), FM966 (*G. hirsutum*), Krasnyj (*G. herbaceum*) and JFW15 (*G. arboreum*). *Arrowheads* point at enlarged region of the CFML in all cotton lines, *arrows* point to CFML bulges paired only in the *G. hirsutum* species. Extra-thickening secondary cell walls are highlighted with a *yellow* line in the JFW15, 25 dpa and mature panels. **b** Enlarged CFML regions (*arrowheads*) displayed variable sizes whereas the CFML bulges (*arrows*) were smaller than 1 μm and were mainly found in pairs in *G. hirsutum*. **c** Paired CFML bulges were also present in *G. hirsutum* ovules cultured in vitro. **d** Longitudinal section of 15 dpa FM966 fibres indicating the three dimensional structure of the enlarged CFML regions (*arrowheads*) and a longitudinal stripe along the fibre in the case of the CFML bulges (*arrow*). An indicated cross section of these fibres following the *yellow* line would show the image equivalent to B. Scale bars =10 μm. All images in a panel are at the same magnification

To study the features of cell walls and middle lamellae during fibre development, resin-embedded transverse sections of fibre tissues from PimaS7 (*G. barbadense*), China10 (*G. barbadense*), FM966 (*G. hirsutum*), Krasnyj (*G. herbaceum*) and JFW15 (*G. arboreum*) were stained with Calcofluor White (Fig. 1). This dye stains a wide range of β-glycans and is commonly used to reveal anatomical and cell wall structures. Staining of fibre sections revealed two distinctive features of the CFML and cell wall interfaces in the fibre tissue: enlarged intercellular regions and smaller, strongly staining, CFML bulges. Intercellular space between cell walls, with content stained with Calcofluor White and wider than 2 μm in diameter, are referred to as enlarged CFML regions and they were present in all cotton species and cultivars analysed, although *G. arboreum* had fewer of them (arrowhead in Fig. 1a). The size of enlarged CFML regions was highly variable within the same tissue and the major axis usually ranged between 2 and 10 μm in transverse sections (arrowheads in Fig. 1b). Additionally, transverse sections of *G. hirsutum* lines presented a remarkably repetitive pattern of two highly staining regions of adjacent fibre cell walls positioned roughly equidistant between cell junctions that were observed throughout the fibre tissue (paired arrows in Fig. 1a and b). These cell wall features were small, 1 μm or less, and the repetitive paired pattern does not appear to have been reported before. FM966 showed abundant paired CFML bulges (arrows in 17dpa FM966 panel) which became apparent at 10 dpa (arrow in 10 dpa FM966 panel) and were also observed at later developmental stages (arrow in 25 dpa FM966 panel). Paired CFML bulges were only evident in FM966*.* While others species also showed occasional single randomly distributed CFML bulges (arrow in 10 dpa PimaS7 panel and in 17 dpa Krasnyj panel), they were not as obvious and structured as in the FM966 line. Paired CFML bulges were also frequently observed in the Coker312 *G. hirsutum* line (Fig. 3A), suggesting that they are a feature of *G. hirsutum* species.

Enlarged CFML regions could be seen in both longitudinal and transversal fibre sections, indicating that they are large three dimensional structures, oval-shaped and filled with particles as stained by Calcofluor White (arrowheads in Fig. 1d). On the other hand, CFML bulges could be followed in serial cross sections indicating that each CFML bulge has the form of a stripe along the fibre as observed in longitudinal sections (arrow in Fig. 1d). Equivalent paired CFML bulges were also present in fibres grown from cultured ovules of FM966 (Fig. 1c) suggesting that their occurrence does not require developing fibres to be inside a locule and, moreover, that the arrangement of paired bulges must be defined by adjacent fibre cells and not by other vegetative parts of the boll.

### The pace of cotton fibre development is determined by cotton genotype

Remarkable differences were observed in the pace of fibre development between the cotton species during five sequential time points post anthesis (5, 10, 17 and 25 dpa and mature fibres) when grown under the same conditions as shown in Fig. 1a. The term *developmental pace* is used here to indicate the speed at which common developmental features, i.e. cell wall adhesion and detachment and secondary cell wall deposition, appeared in different cotton lines. Fibre cell adhesion was obvious by 5 dpa in China10 and FM966 only and enlarged regions of the CFML were already observed in the fibre tissue of these lines (see arrowheads 5 dpa top panels) whereas cell adhesion at the same dpa was incomplete in PimaS7, Krasnyj and JFW15. At 10 dpa all lines showed complete cell adhesion and enlarged regions of the CFML filled with particles were widely present throughout the tissue, except for the JFW15 fibre tissue which showed much fewer enlarged CFML regions. At 17 dpa, the start of cell wall detachment was visible in China10, Krasnyj and JFW15, however fibres in PimaS7 and FM966 were still highly adhered. Cell wall detachment was delayed in the FM966 line compared to the other lines that were observed to start fibre detachment by 17 dpa. Similar cell wall detachment is observed in FM966 only at 25 dpa. Moreover, clear differences in the start and rate of secondary cell wall deposition were also observed. Higher cellulose deposition was seen in the JFW15 line at 17 dpa and, while FM966 fibres showed thinner cell walls compared to any other line at 25 dpa, JFW15 and Krasnyj had extra-thickened cell walls. The higher rate of cellulose deposition in the JFW15 line was very evident at maturation. While the *G. arboreum* (JFW15) and *G. herbaceum* (Krasnyj) cultivars showed an early start to secondary cell wall thickening and higher cellulose deposition rates, the *G. hirsutum* FM966 cultivar showed prolonged cell attachment and specific CFML patterns with a lower cellulose deposition rate than even the *G. barbadense* lines (China10 and PimaS7). Shorter cell adhesion time and a prolonged secondary cell wall deposition in the JFW15 and Krasnyj cultivars may be correlated with lower quality fibres. It is possible that the timing of fibre elongation and the transition phase (that may be linked to cell adhesion and cell detachment) may ultimately influence the degree of maturation and length of cotton fibres.

### CFML dynamics and cell wall glycan composition during fibre development

**Fig. 2:**
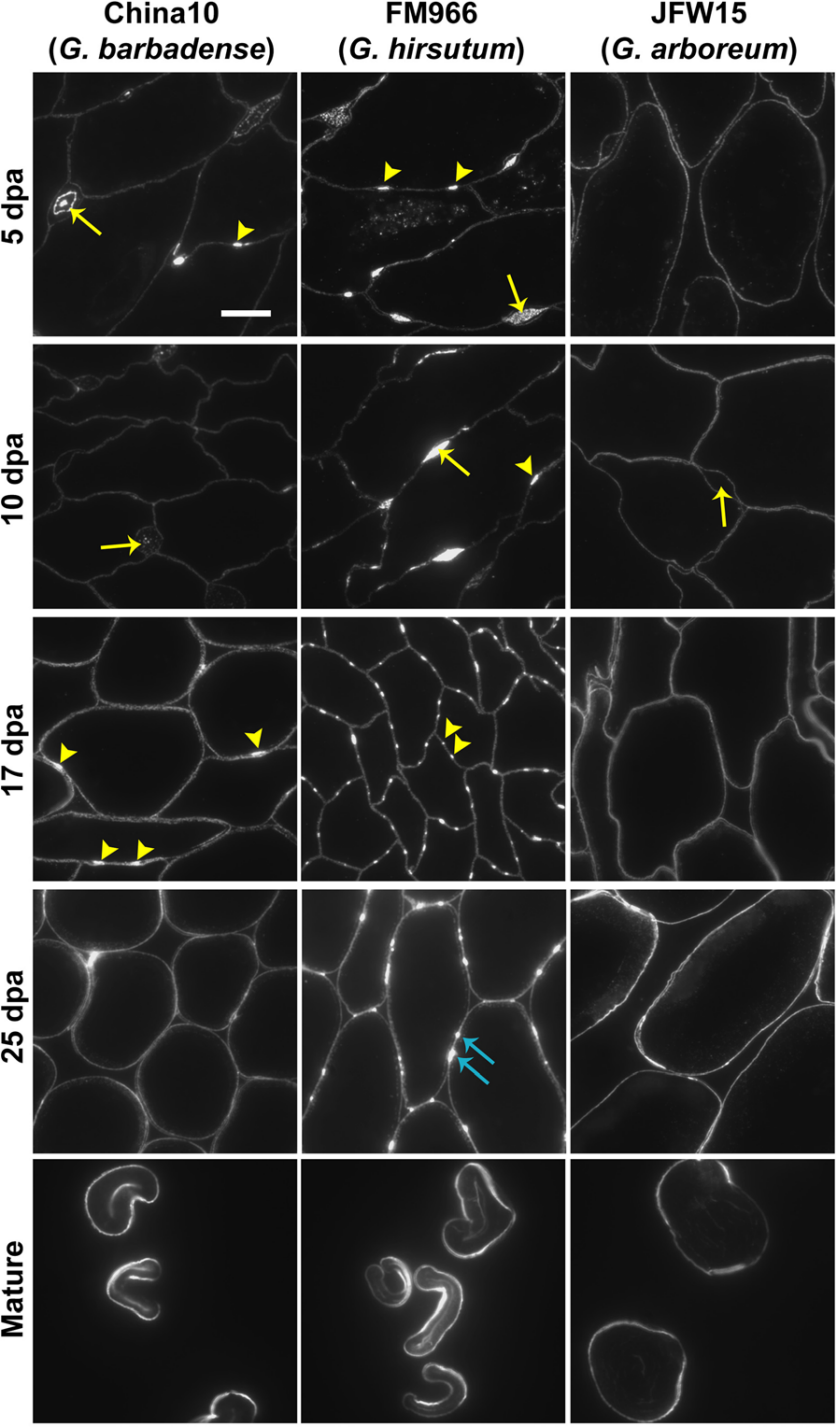
Immunofluorescence labelling of cotton fibre sections with xyloglucan directed mAb LM25. Paired CFML bulges are restricted to the *G. hirsutum* species as labelled by the xyloglucan mAb LM25 in cross sections of fibre tissues at different developmental stages in China10 (*G. barbadense*), FM966 (*G. hirsutum*) and JFW15 (*G. arboreum*) lines. 5 and 10 dpa panels: the LM25 epitope is abundant in the enlarged regions (*arrows*) and bulges (*arrowheads*) of the CFML in the fibre tissue of high quality varieties China10 and FM966 but absent from the JFW15, a low quality fibre variety. 17 dpa panels: xyloglucans are structured in single or paired bulges (*arrowheads*) and remain as anchoring points between cells during cell detachment at 25 dpa in FM966 (*blue arrows*). Scale bar =10 μm. All images in the figure are at the same magnification

Xyloglucans are a major component of the CFML and can be tracked by labelling with the xyloglucan LM25 probe (Fig. 2). LM25-labelled particles were observed at the onset of fibre adhesion and were abundant inside the enlarged CFML regions which are present in the fibre tissue throughout the fibre elongation phase in China10 and FM966 cultivars (arrows in 5 dpa and 10 dpa panels of Fig. 2) and to a much lesser extent in the JFW15 cultivar. Xyloglucan-rich CFML bulges appeared during the elongation phase sporadically as single or paired bulges (arrowheads in 5 and 10 dpa panels of Fig. 2). As fibre development progressed, xyloglucan degradation or rearrangement in the CFML seemed to occur: fewer xyloglucan-rich particles in enlarged CFML regions were observed in favour of abundant xyloglucan bulges between most cells (arrows in 17 and 25 dpa panels of Fig. 2) occurring most abundantly in the FM966 cultivar.

**Fig. 3:**
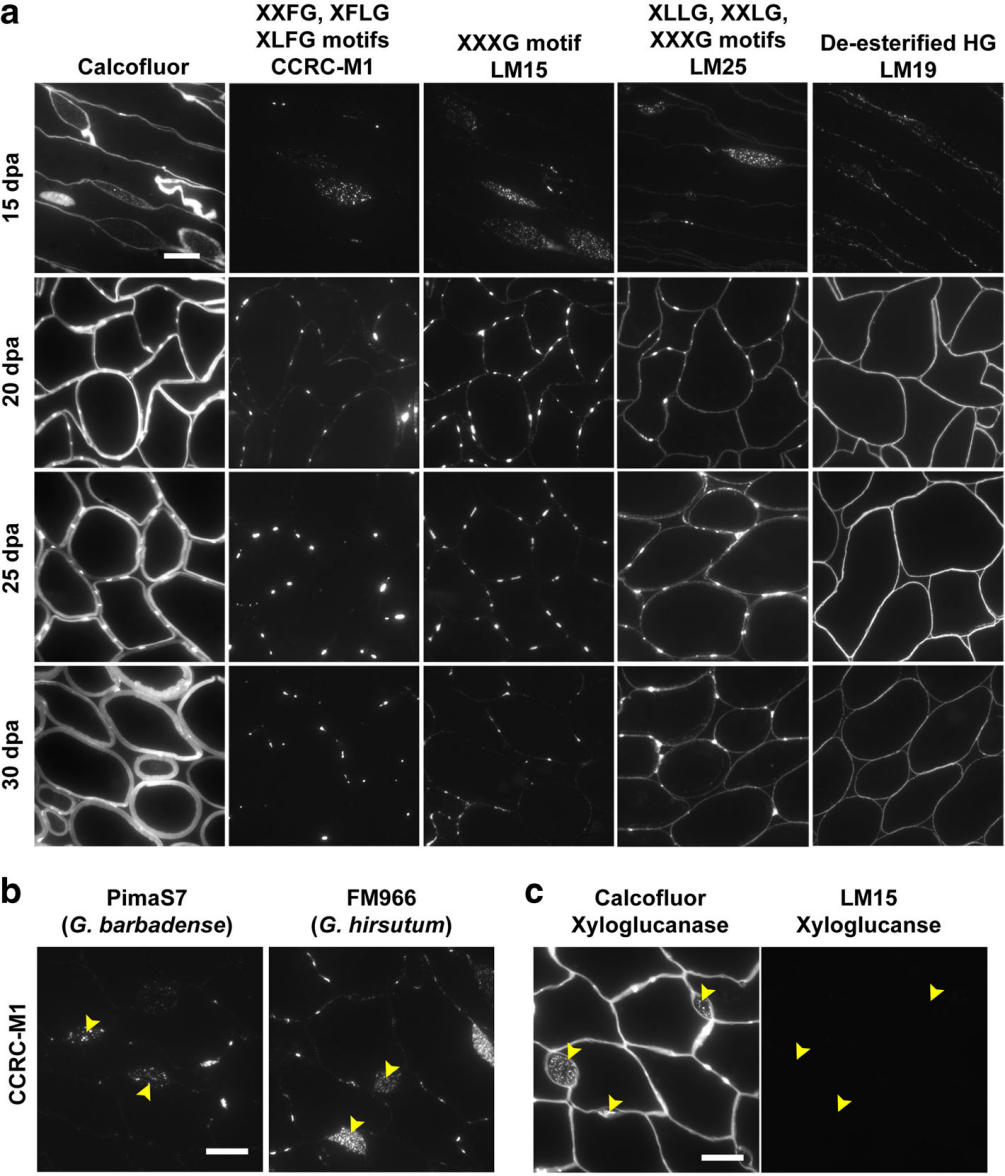
Xyloglucan motifs recognised by CCRC-M1 and LM15 mAbs are specific to the CFML. **a** All micrographs are cross sections of fibre tissue of the Coker variety (*G. hirsutum*). Calcofluor staining of the LM15 labelled sections are shown on the *left-hand* side. The fucosylated and XXXG motifs of xyloglucans (CCRC-M1 and LM15 panels) are prominent in the CFML bulges while these were less abundant in the cotton fibre cell wall where galactosylated xyloglucans appeared homogeneously distributed (LM25 panels). The paired CFML bulges, relevant during the transition phase from elongation to secondary cell wall deposition, are not depicted by the de-esterified HG epitope (LM19 panels). **b** 10 dpa cross sections of the *G. barbadense* PimaS7 and the *G. hirsutum* FM966 cultivars showing the presence of fucosylated xyloglucans in the CFML (*arrowheads*). **c** 15 dpa cross section of the *G. hirsutum* FM966 cultivar treated with xyloglucanase removed xyloglucan (*arrowheads* in *right* panel - LM15) but did not affect the staining of the CFML with Calcofluor White (*arrowheads* in *left* panel) indicating the presence of other β-glucans not characterized yet. Scale bars =10 μm. All images in each panel are at the same magnification

To explore further the composition of the paired bulges of the CFML, cross sections of fibre tissues from another *G. hirsutum* cultivar (Coker312) at 15, 20, 25 and 30 dpa were labelled with a wider range of xyloglucan antibodies (Fig. 3a). The CCRC-M1 and the LM15 probes, along with LM25, showed the particulate nature of the xyloglucan content inside the enlarged region of the CFML and bound strongly to the paired CFML bulges during the beginning of secondary cell wall deposition at 20 and 25 dpa. Cell detachment at cell junctions is evident at 25 dpa while the CFML bulges appear to remain as connecting points between neighbouring cells. Remnants of the CFML bulges were still visible at an advanced secondary cell wall phase (Fig. 3a, 30 dpa). It is of interest that the paired CFML bulges in *G. hirsutum* cultivars also appeared to be anchoring points between cells during the opening of intercellular spaces ahead of complete cell detachment (Fig. 3a, 30 dpa LM25 panels and Fig. 2, 25 dpa FM966 panel).

The CFML from *G. barbadense* cultivars, as analysed [27], was found to differ in composition compared to *G. hirsutum* since fucosylated xyloglucan could not be detected. In our hands, the occurrence of fucosylated xyloglucan in the CFML in *G. barbadense* as well as in *G. hirsutum* cultivars was detected by the CCRC-M1 probe [20] as shown in Fig. 3b. Moreover, treatment of fibre sections with xyloglucanase efficiently removed the xyloglucan LM15 epitope, however it did not abolish Calcofluor White staining (Fig. 3c) suggesting that other β-glycans are present in the CFML currently not detected by any of the available probes.

**Fig. 4:**
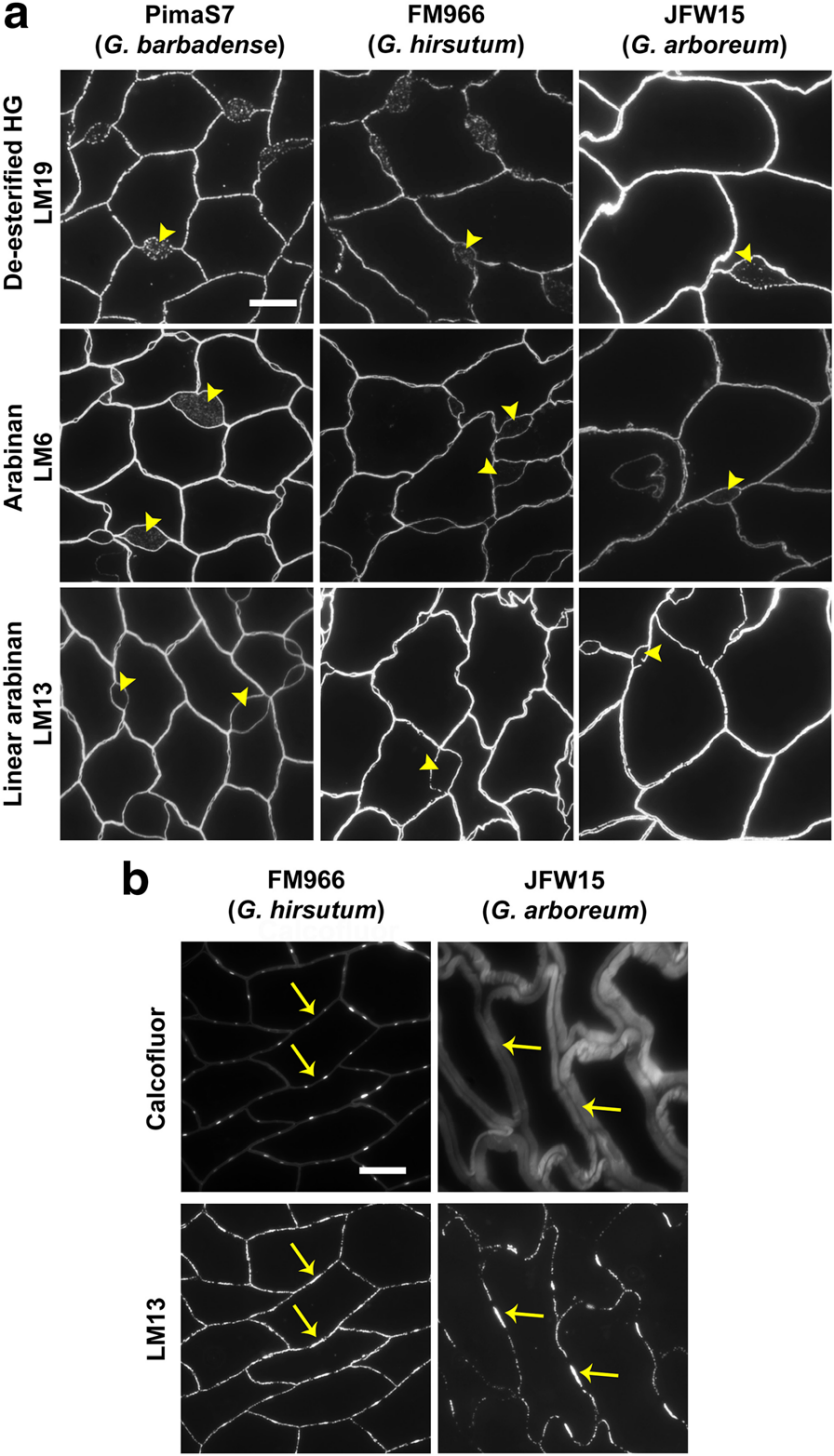
Compositional analysis of the CFML. **a** Cross sections of 10 dpa cotton fibres from PimaS7, FM966 and JFW15 varieties. The de-esterified HG LM19 epitope and the arabinan LM6 epitope are part of the CFML. In contrast, the linear LM13 arabinan epitope labelled all cell walls but was not detected in the enlarged regions and bulges of the CFML. **b** Calcofluor staining and immunofluorescence detection of the linear arabinan LM13 epitope in cross sections of 17 dpa FM966 and JFW15 fibres. Absence of this epitope was observed in the cell wall area around the centre region of two neighbouring cell walls (*arrows*), this area consistently corresponded to cell wall regions between paired CFML bulges in the FM966. Scale bars =10 μm. All images in a panel are at the same magnification

An extended set of antibodies was used to determine the composition of the CFML (Fig. 4). In addition to xyloglucan, the LM19 de-esterified homogalacturonan epitope was also detected inside the enlarged CFML regions (Fig. 4a) in PimaS7 (*G. barbadense*), FM966 (*G. hirsutum*) and JFW15 (*G. arboreum*). In general, the HG LM19 probe showed homogenous labelling inside the enlarged CFML regions, suggesting that the xyloglucan-containing particles could be held in a pectic-based matrix. Similarly to the LM19 epitope, the LM6 arabinan epitope was detected not only in the fibre cell walls but also inside the enlarged CFML regions (Fig. 4a), mostly in the PimaS7 cultivar. On the other hand, the LM13 linear arabinan epitope was not detected in the enlarged CFML regions but strongly bound to cell walls of all cotton lines. In addition, the fibre tissue at the transition phase (17 dpa) in the FM966 *G. hirsutum* line, when the paired CFML bulges are prominent, showed absence of the arabinan LM13 epitope in regions around the CFML bulges (Fig. 4b). Interestingly, a similar sporadic detection of the LM13 epitope was observed between secondary cell walls in JFW15 (Fig. 4b). These observations may indicate a possible role for arabinan degradation as an aspect of cotton fibre cell wall detachment.

## Discussion

Cotton fibres possess a middle lamella that does not originate from cytokinesis after cell plate formation but after the cell-to-cell contact of elongating cotton fibres. The CFML brings fibre cells together and helps them to turn and fold for an optimised packing of cells in the very restrictive volume of the cotton locule during the fast cell elongation phase. Comparison of cell and tissue morphologies in five cotton lines at several dpa, indicated that the timing of cell adherence and the transition phase, as well as the speed at which cellulose is deposited in the secondary cell wall were major morphological variables during fibre development. This work has revealed novel features of the CFML: particle-filled enlarged regions and CFML bulges (mostly paired in the *G. hirsutum* cultivars). Screening with monoclonal antibodies against cell wall polysaccharides revealed the presence of several xyloglucan epitopes including fucosylated xyloglucan (CCRC-M1) in the enlarged CFML regions of both *G. hirsutum* and *G. barbadense* lines as well as arabinan in addition to the previously reported de-esterified HG [4]. In contrast, only xyloglucan was abundantly detected in the CFML bulges.

The origin of the particles in the enlarged CFML regions could be the result of the combined action of several cell wall loosening enzymes such as expansins, endoglucanases and endotransglycosylases that release fragments of the cell wall allowing the cells to expand. The degradation of XG-cellulose tethers is essential in cell wall loosening and this degradation can be done by xyloglucanases and xyloglucan endotransglucosylases/hydrolases (XTH) [28–30]. Literature on cotton fibres has repeatedly reported gene upregulation and peak activities of cell wall remodelling enzymes during fibre elongation which then declines during the transition phase [31–34]. Xyloglucan in the CFML regions could be the result of this extensive enzymatic activity in the cell walls that digest and release polymers into the intercellular spaces to create an adhesive middle lamella. In tomato fruit, xyloglucan has been reported to take part in cell adhesion in the pericarp parenchyma [35]. The observations may indicate a role for the re-modelling of xyloglucan and pectin in the control of fibre cohesiveness.

The paired CFML bulges are perhaps best thought of as linear longitudinal cell wall regions and are a clear case of cell wall heterogeneity. The striking pattern of paired bulges was prominent at the transition phase in FM966 and Coker312 cultivars belonging to *G. hirsutum* species. How the cell regulates the formation of this specific cell wall pattern and for what purpose are intriguing questions. It appears that the CFML organization into a paired pattern is specific to *G. hirsutum* species and although not essential for fibre development, as it is not obvious in other species, these regular structures along elongating axes may somehow be involved in maintaining axial cell elongation. The paired bulges may be linked in some way to the relatively greater elongation rates of *G. hirsutum* lines or alternatively they may perhaps facilitate cell wall detachment at the onset of the phase of secondary cell wall deposition as in several cases they appear to mark the extent of an early stage of intercellular space formation. In this they are reminiscent of the electron dense regions of maize leaf palisade cells during development of mesophyll air spaces [36].

## Conclusion

This work provides the first comprehensive in situ biochemical analysis of the processes of cell adhesion and detachment associated with the CFML that take place during cotton fibre development, and identifies two cell wall features in relation to the CFML; enlarged CFML regions and CFML bulges, the latter with a unique paired pattern in cultivars of *G. hirsutum* species. In conclusion, this work has identified cell wall domains and aspects of cell wall glycan heterogeneity that appear to be linked with fibre cell adhesion and detachment processes which may impact upon fibre quality.
